# A Short Form of the Children’s Experiences of Dental Anxiety Measure (CEDAM): Validation and Evaluation of the CEDAM-8

**DOI:** 10.3390/dj9060071

**Published:** 2021-06-15

**Authors:** Jenny Marie Porritt, Annie Morgan, Helen Rodd, Fiona Gilchrist, Sarah R. Baker, Tim Newton, Zoe Marshman

**Affiliations:** 1 Department of Psychology, Sociology, and Politics, Sheffield Hallam University, Sheffield S10 2BQ, UK; 2 Paediatric Dentistry Department, Charles Clifford Dental Hospital, Sheffield S10 2SZ, UK; a.g.morgan@sheffield.ac.uk; 3 School of Clinical Dentistry, University of Sheffield, Sheffield S10 2TA, UK; h.d.rodd@sheffield.ac.uk (H.R.); f.gilchrist@sheffield.ac.uk (F.G.); s.r.baker@sheffield.ac.uk (S.R.B.); z.marshman@sheffield.ac.uk (Z.M.); 4 Oral Health Services Research and Dental Public Health, King’s College London, London SE1 9RT, UK; tim.newton@kcl.ac.uk

**Keywords:** dental anxiety, children, child-centred, short-form, assessment

## Abstract

Background: The Children’s Experiences of Dental Anxiety Measure (CEDAM-14) is a child-centred measure of dental anxiety which assesses a range of behaviours, thoughts and feelings/physical symptoms related to dental anxiety. A short form of the CEDAM-14, which places less time burden on patients and clinicians, could promote the feasibility and applicability of the CEDAM in clinical settings. The aim of the study was to develop a short version of the CEDAM that can be used to assess children’s dental anxiety in clinical practice. Methods: A short version of the CEDAM was developed using a combination of item impact and regression methods. Measurement properties including floor/ceiling effects, variance, criterion validity, construct validity and internal consistency was calculated for the short form. Results: An eight-item CEDAM short form was developed (CEDAM-8) that had good psychometric properties, was significantly correlated with the CEDAM measure (r = 0.90; *p* < 0.01), had minimal floor and ceiling effects (3.5% and 1.2%, respectively) and was sensitive to change. Conclusion: The CEDAM-8 is a useful assessment tool for clinicians that is easy and quick to administer and could help to understand children’s experiences of dental anxiety and changes in anxiety over time and following intervention.

## 1. Introduction

In order to accurately and meaningfully assess children’s dental anxiety it is important that dental anxiety measures are informed by theoretical frameworks of the phenomenon of interest (dental anxiety) and that they actually assess the experiences/symptoms that matter most to children [1]. The Children’s Experiences of Dental Anxiety Measure (CEDAM-14) was therefore developed as a child-centred measure of dental anxiety [2] for children aged 9–16 years. It was derived from interviews with children [3] and based on a Cognitive Behaviour Therapy (CBT) assessment model of dental anxiety [4]. The CEDAM-14 includes items designed to assess the behavioural, cognitive and physical symptoms/feelings that maintain children’s dental anxiety (e.g., lack of perceived control, low levels of trust, behavioural avoidance), which could be discussed with the patient and addressed in order to effectively manage the individual’s anxiety. The CEDAM-14 has good psychometric properties and has proved suitable for use in research, clinical settings and health care evaluations [2,5]. However, only a small proportion of dentists incorporate the use of validated scales into patient assessments [6]. Research has found that the majority of dentists instead rely on their own judgement to assess a child’s anxiety [7]. In addition, the perceived burden completion of a measure places on the child, the time constraints and the lack of experience in administering anxiety questionnaires are all barriers identified in the literature [7]. It has been recommended that if measures for children’s dental anxiety are to be incorporated into routine assessments, they need to be brief, straightforward and require no additional scoring [7]. The CEDAM-14 was validated using a transformed interval scoring system and whilst transformed scores can be advantageous for measuring change between/within groups in research studies, this additional scoring could actually be a barrier to its widespread use in clinical practice.

Developing a short form of the CEDAM-14 measure which does not require additional scoring could therefore promote the use of this measure in clinical practice. There are no guidelines for creating short forms, or for the minimum required number of items that should be included. However, there are a variety of methods which can be used to develop these questionnaires and short forms with as few as six items have been shown to demonstrate high levels of validity and reliability [8,9]. The use of statistical techniques alone to develop short forms has been criticised [8] and it has been proposed that researchers should take both theoretical and statistical considerations into account [10]. The item impact method is an approach which can be useful in situations where investigators want to keep items that are important to patients [11] and thus would be particularly suitable for measures such as the CEDAM, which aim to be child-centred. Developing short forms which have high internal consistency but also sufficient breadth is also important [10]. The development of the original CEDAM-14 was underpinned by theory and therefore it is equally important that this theoretical framework guides the development of the CEDAM short form and that the breadth of what the measure is assessing is not compromised (e.g., it still assesses behaviour, thoughts and physical symptoms/feelings).

For clinical assessment tools to be useful it is also important that they are able to capture meaningful changes in what they are measuring [12]. Development of a short form that is unable to detect changes in dental anxiety, where changes would be expected, would reduce the usefulness of the measure. There is no gold standard for how to identify whether a minimal important difference (MID) has occurred using a given measure, and a range of distribution and anchor-based methods have been used to help researchers or clinicians determine MIDs for a measure [13,14]. However, anchor-based approaches are most commonly used for establishing a MID score for measures and these often use a global item that assesses meaningful change from the perspective of the patient or practitioner as an anchor [14]. 

The aim of the current study was to develop a short form of the CEDAM-14 based on statistical and theoretical considerations. Preliminary analysis, using an anchor-based approach, was also conducted to determine the MID for the CEDAM short form. 

## 2. Methods

Data were obtained from the clinical and non-clinical samples that developed and validated the original CEDAM (*n* = 247) (study 1, previously published [2]) and were combined with CEDAM-14 data reported by children aged between 9–16 years old who had attended a large UK Paediatric Dental Hospital for new patient and follow-up appointments between the dates of July 2016 and January 2017 (study 2, *n* = 99, 39% male, mean age = 11.1 years (SD = 2.0), data not published). Participants were recruited to test a new web-based app version of the CEDAM. Children were included if they had a clinical diagnosis of dental anxiety, based on a subjective report from the dental professional responsible for their management, and were able to complete both paper and electronic CEDAM versions. Only results from completion of the paper version of the CEDAM-14 in study 2 were included in this data set.

Ethical approval for both studies was obtained prior to study commencement and written informed assent from children and consent from parents were obtained in all instances (ethical approval codes: 13/YH/0163 and 16/YH/0038). The combined data set (*n* = 346, 40% male, mean age 12.2 years (SD = 1.8)) was split into two equal halves at random (*n* = 173) and the short form was developed using the first data set (mean age 12.3 years (SD = 1.8), 45% male) and data were evaluated using the second data set (mean age 12.1 years (SD = 1.8), 35% male), which follows methods previously developed to derive short forms [15]. The sample size in this study is comparable to previous research that has successfully validated short forms using a similar approach [15]. Four individuals had partial missing CEDAM data (range 1–10 missing data points) and total CEDAM scores were not calculated for these individuals. However, data for these individuals were included for the purposes of detailing which CEDAM items had missing data. Statistical tests were undertaken using SPSS (v24 IBM Corp., Armonk, NY, USA).

### 2.1. Development

In line with previous research [15,16] item impact and regression methods were employed to develop the CEDAM-8. The item impact method enables the researcher to identify the items which are most important to participants and involves multiplying the frequency of individuals experiencing an item by the item’s mean score [17]. Therefore, the item impact scores for each CEDAM question were calculated by multiplying the proportion of participants indicating they experienced that impact/anxiety (scores of 2 or 3) with the mean sample score for that question. Items were ranked based on their item impact score and the top six highest ranking items were selected for possible inclusion in the CEDAM short form. 

Using the regression technique, the transformed interval total score for the original 14-item CEDAM was used as the dependent variable and all of the individual items entered as independent variables and single multiple regression analysis conducted. Items were ranked by their contribution to the coefficient of variance (R^2^) and the six items which made the largest contribution were selected for possible inclusion in the CEDAM short form.

### 2.2. Evaluation

Face validity of the proposed short form was assessed by the multidisciplinary team (which included health psychologists, senior clinical academics in paediatric dentistry and dental public health). Whilst the CEDAM-14 is a unidimensional measure it was developed using the Five Areas CBT model to capture a variety of thoughts, feelings/physical symptoms and behaviours which children felt were central to their dental anxiety [3]. Therefore, the team felt it was important, from a theoretical perspective, that any short form captures a range of behaviours, thoughts and feelings/physical symptoms experienced by children. At this initial stage, the short form was also assessed for missing data. 

Using the second data set the short form was subject to statistical analysis. The short form was calculated by totalling the scores for each item included in the measure. Content validity was assessed through examination of descriptive statistics and examination of floor and ceiling effects for the measure. Internal consistency reliability was determined using Cronbach’s alpha. Alphas were calculated with each item deleted and corrected item total correlations. Criterion validity was tested by correlating the short form against the CEDAM-14. Construct validity involved correlating the short form with a global item measuring how scared children felt about visiting the dentist, which was scored on a 4-point scale (1 = I don’t feel scared at all, 2 = I feel a little scared, 3 = I feel quite scared, 4 = I feel really scared).

Responsiveness of the CEDAM-8 short form was assessed using data from the sample of young people who had received a CBT-based intervention to reduce their dental anxiety (*n* = 38) and the ability of the short measure to detect changes in dental anxiety following this anxiety-based intervention was tested. In addition to examining the short form’s ability to detect reductions in anxiety over time, where changes would be expected, the MID score was investigated. The MID is the minimum reduction in score that results in benefits to the patient, clinical team or has implications for the patient’s treatments [18]. An anchor-based approach was used to calculate the MID score for the short form. A global item measuring overall changes in dental anxiety, from the patients’ perspective, was used as the anchor (‘Has how you feel about going to the dentist changed since you started using the green guide?’). The global item was scored on a 5-point scale (1 = I feel a lot less worried, 2 = I feel a little less worried, 3 = my feelings have not changed, 4 = I feel a little bit more worried, 5 = I feel a lot more worried). Two groups were created based on their responses to the global item. Those who indicated there had been no change or little change (scored 2 or 3) were categorised into the no MID group and those who indicated they felt a lot less worried (scored 1) were categorised into the MID group. No participants indicated they felt more worried (scored 4 or 5). The change difference (CD) score was then calculated, which is the most widely used analytical approach to calculate a MID [14]. This method focuses on the CEDAM score change of those who responded in the desired way on the anchor (e.g., patient reported a MID). Identifying a measure’s MID score is important for intervention evaluation [19,20].

## 3. Results

### 3.1. Development of the Short Form Measure

The short form was developed using the first half of the data set (*n* = 173) using item impact and regression methods. Descriptive information for the item impact and regression analyses can be seen in Table 1. The item impact and regression methods identified four common items within the top six ranking items from each approach (feeling worried about the need to have something done, thinking it will be painful, feeling stressed, feeling shaky). These four common items and the two of the additional highest-ranking items from each method were combined to create an eight-item short form (CEDAM-8).

### 3.2. Evaluation of the Short Form Measure 

The CEDAM-8 included a mixture of items assessing behaviours (2 items), thoughts (3 items) and physical symptoms/feelings (3 items) and no issues were identified in relation to face validity. The second half of the data set (*n* = 173) was used to evaluate the short form and the psychometric properties of the CEDAM-8 can be seen in Table 2. The short form had good variance/range of scores and internal consistency (Cronbach’s alpha 0.86 and alphas remained stable when items were deleted). The CEDAM-8 had no floor or ceiling effects (3.5% and 1.2%, respectively, which falls within the acceptable range of <15%). The short form correlated highly with the CEDAM-14 (r = 0.90) indicating excellent criterion validity and had good construct validity (correlated highly with the global dental anxiety item, r = 0.77).

### 3.3. Responsiveness

Responsiveness was examined using pre- and post-intervention data (*n* = 38) and revealed that the CEDAM-8 was able to detect significant reductions in dental anxiety over time, in response to an intervention designed to reduce dental anxiety (see Table 3). 

Preliminary analysis of the mean change score required to make a meaningful difference to children’s anxiety levels (MID) revealed that patients who reported only a little reduction in worry had mean reductions of −3.23 and −2.5 for the CEDAM-8 (possible range 8–24) and CEDAM-14 forms (possible range 14–42), respectively. Children who indicated that they had experienced a lot of reduction in their worry had a mean score change of 3.9 for the CEDAM-8 and CEDAM-14. This preliminary analysis would suggest that an improvement of at least 3.9 points on either the short form or original CEDAM could be considered to be the minimal important change which is relevant/meaningful from the patient’s perspective. 

## 4. Discussion

The original CEDAM measure (CEDAM-14) was developed with children and was based on a CBT assessment model of dental anxiety with the aim of assessing experiences/symptoms which influence and maintain children’s dental anxiety over time [2,4]. The original CEDAM measure was unidimensional but included a broad range of experiences based on the CBT model used [4], such as unhelpful thoughts, behaviours and physical symptoms/feelings. Short forms can broaden the application of a measure by reducing the amount of time and costs associated with assessment and the risk of missing items [8,21]. 

A short form of the CEDAM-14 was therefore developed using alternative statistical techniques (the item impact and regressions methods) and consideration of the theoretical framework which guided the original measure. The six highest ranking items using each method identified four common items which were included in the short form (worrying about needing something done, thinking it will be painful, feeling stressed, feeling shaky). The item impact methodology also identified items relating to perceived control and avoidance as additional high-ranking items and the regression methodology identified feeling upset and talking to parents about not wanting to visit the dentist, as additional high-ranking items. There is significant support within the literature that these additional items are important factors in the development and maintenance of dental anxiety and thus a common goal of interventions is to reduce avoidance behaviours [22,23]. It has been argued that neither the item impact or regression methodology are inherently superior and that it is the content and properties of the respective measure which are most important [15]. Therefore, the four common items and four additional high-ranking items (two highest ranking items from each method) were incorporated into the development of an eight-item short form (CEDAM-8). 

The CEDAM-8 includes of mixture of important behavioural, cognitive and physical symptoms/feelings related to how children experience dental anxiety and thus assesses the complex multifaceted experience of dental anxiety. It is important that both the patient and members of the dental team understand the factors which may be maintaining the child’s anxiety, and examination of the CEDAM-8 scores can help provide a starting point for discussing how the child’s specific thoughts or behaviours may be contributing to their anxiety and how they will be addressed by the dental team. For example, if a child indicates on the measure that they feel they have low perceived control this could be a specific area where the team could offer the child support (e.g., implement strategies which enhance control). If the child is struggling with the physiological symptoms of anxiety the dental team may focus on teaching the child some relaxation strategies. Indeed, the CBT assessment model, used to develop the measure, aims to provide clinicians and patients with information about what factors could be targeted to reduce anxiety [4]. The examination of which behaviours, thoughts or symptoms improve (or worsen) over time and/or in response to treatment could be useful for clinicians and feed into the future management of the child’s anxiety. There is also evidence that just the process of the patient communicating their anxiety to their dental team can decrease their feelings of anxiety [24]. The CEDAM-8 could therefore act as a valuable clinical tool that can aid effective communication and promote collaboration between the child, parent and dental team. Positive patient-dentist interactions are fundamental to the delivery of patient-centred care and play an important role in promoting regular dental attendance [25]. 

The CEDAM-8 had good psychometric properties. Presence of floor or ceiling effects indicate problems with content validity and previous research has used the threshold of <15% patients with minimum/maximum scores as acceptable [18]. The CEDAM-8 short form did not have problematic floor or ceiling effects (3.5% and 1.2%, respectively), had good internal consistency and correlated highly with the original CEDAM and global ratings of dental anxiety, demonstrating good construct and criterion validity. Research has found that dental professionals are reluctant to use dental anxiety measures that require additional scoring [7]. The CEDAM-14 was validated using transformed scores and thus it is recommended that transformation of scores be undertaken when this form is used in research to detect change. Whilst this may not be a problem in research studies, where additional time for analysis is often available, it could prevent clinicians from using the measure in clinical practice. The CEDAM-8 does not require transformation of raw scores in order to detect change and therefore overcomes this potential barrier. It is recognised that advantages improving application of a measure, through the development of a short form, can come at the cost of the measures properties and all statistical methods used to reduce the length of measures have their own set of limitations. Future research needs to undertake further testing of the CEDAM-8 using independent and diverse samples, as recommended within the literature [8]. 

This work provides the first estimates of a MID for a child-centred dental anxiety measure. This has important relevance to clinicians and researchers as it allows them to understand what constitutes meaningful change. Some measures of dental anxiety (mainly adult measures) have provided thresholds for mild, moderate and severe anxiety, which is useful for appreciating the severity of an individual’s anxiety and informing subsequent treatment decisions (e.g., whether it is appropriate to refer a patient to a particular service). However, it can also be useful to know whether an intervention has resulted in meaningful change to that patient. An anchor-based method was used to obtain preliminary evidence for a MID change score for the CEDAM-8 short form. In the current study the MID was calculated using a global item which measures change from the patient’s perspective. It has been suggested that for measures which assess aspects of wellbeing then the patient’s perspective must be key in the development of the MID, as opposed to measures assessing disease where clinician judgement could be more useful [13,26]. The findings suggested a score reduction of 3.9 for the CEDAM-8 could result in a change which is meaningful from the patient’s perspective. Awareness of a measure’s MID score can help clinicians develop knowledge of whether interventions have effectively reduced patients’ dental anxiety levels and can also be useful when planning intervention studies and clinical trials (e.g., to inform sample size calculations). 

The scrutiny of a measure’s MID is incredibly important, however, and caution should be applied to these findings given the limited sample size. Cook [27] highlights how researchers or clinicians may condemn an intervention because a group failed to meet the MID when actually the MID they are using is indeed problematic. Further testing of the MID for the CEDAM-8 (and CEDAM-14) is therefore required. Whilst there is not a consensus for the standardised methodology that should be used for calculating the MID one of the criticisms of the anchor-based technique using a patient perspective is that recall bias could influence an individual’s memory of ‘change’ [12]. MID scores could also be established based on ‘meaningful’ change as assessed by the dental professional (e.g., fewer difficulties/challenges encountered when managing the patient’s care) or based on change which has implications for the patient’s treatment (e.g., need for sedation/general anaesthetic). The use of multiple, independent anchors would help address the issue of recall bias and enable a range of MID scores to be established, which are based on different types of anchors [14,28]. 

Whilst there is no gold standard for how short forms should be developed, there are a series of methodological and statistical issues which should be considered when shortening questionnaires and all methods available have their own set of limitations [8,29]. In the current study, the short form was developed using some of the data that was used to validate the original measure and it is important to recognise that this could have resulted in higher estimates of agreements between the two forms [10]. However, the two-phase design used in the current study adhered to recommendations in the literature which suggests that two independent samples be used for the shortening process (selecting the items) and the subsequent validation phase [8]. Additionally, the data obtained to develop the short form in the current study was largely taken from cross-sectional research undertaken in the U.K. (data from one small scale intervention study was included) and therefore it will be important to further validate the measure using a more advanced study design with diverse populations. Finally, whilst short forms are more feasible and practical for clinical use, long forms are able to assess a wider range of experiences, and thus can be superior for capturing more in-depth experience of the phenomenon, which may make these original measures more suitable for research purposes. 

The CEDAM-8 is a freely available, valid and reliable child-centred measure of dental anxiety that can be used by clinicians and/or researchers interested in children’s experiences of dental anxiety to capture how an individual is experiencing dental anxiety and how their anxiety changes over time and in response to an intervention. It can be used with children aged between 9–16 years old and in conjunction with the ‘Your Teeth You are in Control’ guide [30] to measure changes in young people’s anxiety levels following this CBT-based assisted self-help intervention. Given this intervention has been translated into multiple languages it is important that this measure of dental anxiety also be evaluated and validated in other languages. Future work should focus on addressing the additional barriers that are purported to prevent clinicians from undertaking patient-centred assessments (e.g., the perceived lack of benefit of formal anxiety assessment) [7] to ensure that clinicians are supported in the assessment of dental anxiety within routine care.

## 5. Conclusions

The CEDAM-8 is a brief measure of dental anxiety which can be used to assess children’s anxious thoughts, behaviours and physical symptoms/feelings. The use of this measure in clinical practice could act as a valuable communication aid and provide dental teams with a quick and easy way of gaining insight into their patient’s concerns and worries, developing understanding of the factors that are maintaining the patient’s anxiety. Assessment of dental anxiety using the CEDAM-8 enables the child, parent and dental team to work together to break the vicious cycle of anxiety. The measure has shown responsiveness to change and therefore dental teams and services may also use the CEDAM-8 to demonstrate evidence of effective patient-centred management of dental anxiety within their practice.

## Figures and Tables

**Table 1:** Short form analysis (data set 1, *n* = 173).

CEDAM-14 Item	Proportion Experienced Impact	Mean Score	Item Impact Score	Item Impact Rank	R^2^	Regression Analysis Rank	Top Six Items from Item Impact Analysis	Top Six Items from Regression Analysis
1. Avoidance (B)	51.4%	1.7	87.4	5	0.41	8	/	
2. Talk to parents about whether want to go (B)	28.3%	1.4	39.6	8	0.48	3		/
3. Let the dentist look in mouth (B)	9.9%	1.1	10.9	14	0.30	10		
4. Worry if need to have something done (T)	72.8%	1.9	138.3	2	0.53	2	/	/
5. Think they will stop if asked (T)	35.8%	1.4	50.1	7	0.20	13		
6. Think it will be painful (T)	66.4%	1.8	119.5	3	0.44	6	/	/
7. Think things could go wrong (T)	26.6%	1.3	34.6	11	0.43	7		
8. Think will have control over what happens (T)	74.0%	1.9	140.6	1	0.26	12	/	
9. Feel shaky (P/F)	56.1%	1.7	95.4	4	0.47	4	/	/
10. Feel stressed (P/F)	44.5%	1.5	66.8	6	0.55	1	/	/
11. Feel upset (P/F)	27.8%	1.3	36.1	10	0.45	5		/
12. Feel embarrassed (P/F)	28.9%	1.3	37.6	9	0.37	9		
13. Feel angry (P/F)	13.9%	1.2	16.7	13	0.30	10		
14. Feel trust (P/F)	18.5%	1.2	22.2	12	0.28	11		

B = behaviours; T = thoughts; P/F= physical symptoms or feelings. 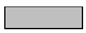 Items which ranked in the top six from the item impact and/or regression analysis.

**Table 2:** Psychometric properties of the CEDAM-8 (data set 2, *n* = 173).

CEDAM-8 Form	Amount of Missing Data	Cronbach’s Alpha	Range of Alphas If Items Deleted	Range of Corrected Item Total Correlations	Mean Score (SD)	Range of Scores	% of Children with Minimum (8) to Maximum Scores (24)	Construct Validity (Global Dental Anxiety Item)	Criterion Validity (CEDAM-14)
**CEDAM-8** Avoidance (B) Talk to parents (B) Worry if need to have something done (T) Think it will be painful (T) Think will have control over what happens (T) Feel shaky (P/F) Feel stressed (P/F) Feel upset (P/F)	*n* = 1	0.86	0.83–0.86	0.40–0.69	13.65 (3.74)	8–24	Min: 3.6% (*n* = 7) Max: 1.2% (*n* = 2)	r = 0.77 (*n* = 84) *p* < 0.01	r = 0.90 (*n* = 170), *p* < 0.01

B = behaviours; T = thoughts; P/F = physical symptoms or feelings.

**Table 3:** Responsiveness of CEDAM measures.

	Mean Score pre and Post CBT Intervention (SD)	Mean CEDAM Score Change (SD): No Change/a Little Less Worried Group	Mean CEDAM Score Change (SD): A lot Less Worried Group
CEDAM-14	Pre-CBT: 22.36 (2.57) (*n* = 38) Post CBT: 18.88 (2.42) (*n* = 38) T = 9.54 (df 37), *p* < 0.01	Mean change: −2.47 (1.66) *n* = 13	Mean change: −3.86 (2.29) *n* = 24
CEDAM-8	Pre-CBT: 15.42 (3.05) (*n* = 38) Post CBT: 11.68 (2.46) (*n* = 38) t = 8.44 (df = 37), *p* < 0.01 (95% CI: 2.84–4.63)	Mean change: −3.23 (2.59) *n* = 13	Mean change: −3.88 (2.80) *n* = 24

## Data Availability

Please contact the corresponding author with data requests.
